# Changes in the structure, perfusion, and function of the hippocampus in type 2 diabetes mellitus

**DOI:** 10.3389/fnins.2022.1070911

**Published:** 2023-01-09

**Authors:** Mingrui Li, Yifan Li, Kui Zhao, Xin Tan, Yuna Chen, Chunhong Qin, Shijun Qiu, Yi Liang

**Affiliations:** ^1^The First Clinical Medical College, Guangzhou University of Chinese Medicine, Guangzhou, China; ^2^Department of Radiology, The First Affiliated Hospital of Guangzhou University of Chinese Medicine, Guangzhou, China; ^3^Department of Magnetic Resonance Imaging, Zhanjiang First Hospital of Traditional Chinese Medicine, Zhanjiang, China

**Keywords:** type 2 diabetes mellitus, hippocampus, volume, cerebral blood flow, resting-state functional MRI

## Abstract

**Objective:**

This study aims to explore the changes in the structure, perfusion, and function of the bilateral hippocampus in type 2 diabetes mellitus (T2DM) applying multimodal MRI methods, hoping to provide reliable neuroimaging evidence for the diagnosis of hippocampus-related brain injury in T2DM.

**Methods:**

We recruited 30 T2DM patients and 45 healthy controls (HCs), on which we performed 3D T1-weighted images, resting-state functional MRI (rs-fMRI), arterial spin labeling (ASL) sequences, and a series of cognitive tests. Then, we compared the differences of two groups in the cerebral blood flow (CBF) value, amplitude of low-frequency fluctuation (ALFF) value, fractional ALFF (fALFF) value, coherence-based regional homogeneity (Cohe-ReHo) value, and degree centrality (DC) values of the bilateral hippocampus.

**Results:**

In the T2DM group, the bilateral hippocampal volumes and the CBF value of the right hippocampus were lower than those in the HCs, while the ALFF value, fALFF value, and Cohe-ReHo value of the bilateral hippocampus were higher than those in the HCs. Correlation analysis showed that fasting blood glucose (FBG) was negatively correlated with the residuals of left hippocampal volume (*r* = −0.407, *P* = 0.025) and right hippocampal volume (*r* = −0.420, *P* = 0.021). The residual of the auditory-verbal learning test (AVLT) (immediate) score was positively correlated with the residual of right hippocampal volume (*r* = 0.369, *P* = 0.045).

**Conclusion:**

This study indicated that the volume and perfusion of the hippocampus are decreased in T2DM patients that related to chronic hyperglycemia. Local spontaneous neural activity and coordination are increased in the hippocampus of T2DM patients, possibly as an adaptive compensation for cognitive decline.

## 1. Introduction

Type 2 diabetes mellitus (T2DM) poses a great threat to human health and creates a significant socioeconomic burden (Sun et al., 2022). Cerebrovascular diseases caused by chronic hyperglycemia result in decreased cerebral blood flow (CBF), and ischemia and hypoxia can affect the function of nerve cells and ultimately cause them to undergo apoptosis, thus affecting patients’ cognitive function. Moreover, insulin resistance is an important cause of cognitive decline. The action of insulin on the central nervous system is complex. Abundant insulin receptors are present in both astrocytes and neuronal synapses in brain, and insulin signaling can be conductive to the regulation of synaptogenesis and remodeling (Abbott et al., 1999). In addition, insulin exerts other neuromodulator effects that affect cognition, such as affecting cognitive function by regulating the expression of the neurotransmitters acetylcholine and norepinephrine (Figlewicz et al., 1993; Kopf and Baratti, 1999) and increasing glucose metabolism in the cerebral cortex, which is involved in learning and memory (Bingham et al., 2002).

The hippocampus is one of the main components of the limbic system, which is involved in advanced cognitive functions. Abundant insulin receptors are present in the hippocampus (Schulingkamp et al., 2000), and insulin signaling is closely related to structure and function of hippocampus. Thus, the hippocampus is more susceptible to central insulin resistance than other brain regions (Biessels and Reagan, 2015; Kullmann et al., 2020). Furthermore, the hippocampus is a relatively fragile region. Previous studies have shown that it is more vulnerable to various injuries, such as severe hypoxia (Cervos-Navarro and Diemer, 1991; Mcewen, 1997). Therefore, the hippocampus may be an important target of central damage secondary to T2DM. According to previous studies, cognitive impairment in T2DM patients linked to changes in hippocampal structure and function (Hu et al., 2019; Li et al., 2020). Xia et al. (2020), using Granger causality analysis with hippocampus as a region of interest (ROI), revealed that T2DM patients without mild cognitive impairment (MCI) had abnormal hippocampal directional connectivity compared to healthy controls (HCs). The major abnormalities were located in the prefrontal cortex, cingulate cortex, and amygdala, all of which are belong to the Papez circuit. This altered network connectivity is associated with insulin resistance.

In this study, we hypothesized that the structure, perfusion, and function of the hippocampus in T2DM patients were changed, and these changes were associated with the decline of cognitive function. Therefore, we analyzed the changes in the structure, perfusion, and function of the bilateral hippocampus in T2DM patients by multimodal MRI imaging and to analyze their correlation among imaging indicators, clinical laboratory indicators and neurocognitive test scores. We aimed to explore the underlying neural mechanisms of hippocampal-related brain injury in T2DM and its multimodal MRI imaging biomarkers, to provide reliable neuroimaging evidence for the diagnosis of hippocampus-related brain injury in T2DM.

## 2. Materials and methods

### 2.1. Participants

Participants were enrolled from the First Affiliated Hospital of Guangzhou University of Chinese Medicine. All participants were right-handed, native Chinese speakers of Han ethnicity. The inclusion and exclusion criteria were the same as those in our previous study (Li et al., 2021). Thirty T2DM patients and 45 HCs were included.

### 2.2. Clinical data

Clinical indicators for T2DM patients include glycated hemoglobin (HbA1c), fasting blood glucose (FBG), and fasting insulin (FINS). The homeostatic model assessment for insulin resistance (HOMA-IR) was calculated by HOMA-IR = FBG × FINS/22.5.

### 2.3. Cognitive evaluation

Multifaceted cognitive assessments were performed which contain the Montreal cognitive assessment (MoCA), auditory-verbal learning test (AVLT), digit span test (DST), clock-drawing test (CDT), and grooved pegboard test (GPT).

### 2.4. MRI data

The MRI data were obtained on a 3T GE SIGNA MRI scanner with an 8-channel phased-array head coil. T1-weighted, T2-weighted, and T2 fluid-attenuated inversion recovery images were acquired to exclude organic brain lesions. Then resting-state functional MRI (rs-fMRI) scans (TR = 2,000 ms, TE = 30 ms, flip angle = 90°, slice thickness = 3 mm, gap = 1 mm, FOV = 220 mm × 220 mm, Matrix = 64 × 64, slices = 36, measurements = 185) and high-resolution 3D T1-weighted images scans (TR = 8.15 ms, TE = 3.17 ms, flip angle = 12°, slice thickness = 1 mm, FOV = 256 mm × 256 mm, Matrix = 256 × 256, slices = 188, NEX = 1) were performed successively. At the same time, we performed an arterial spin labeling (ASL) scan using imaging parameters: TR = 5,007 ms, TE = 10.4 ms, NEX = 3, slice thickness = 3 mm, FOV = 240 mm × 240 mm, recon matrix = 128, arms = 8, acquisition points = 512, slices = 50.

### 2.5. Small-vessel disease assessment

The Wahlund scoring rules assessing changes in white matter were used on the T2-FLAIR images (Wahlund et al., 2001). Five regions were then quantitatively assessed for white matter hyperintensities and lacunar infarcts, including bilateral frontal, parietal and occipital, temporal, cerebellar and brainstem, and basal ganglia. Participants with a rating score > 2 were excluded. The ratings were assigned independently by two physicians with imaging experience who were blinded to the group assignments. If there was any disagreement between the two physicians, the evaluation were conducted jointly.

### 2.6. Image processing

We used FreeSurfer software version 6.1 to calculate bilateral hippocampal volume for each subject from 3D T1-weighted images, and to reduce the effect of individual differences, we simultaneously considered the estimated total intracranial volume (eTIV) as a covariate. The CBF map for each subject was calculated from the 3D-ASL images using the post-processing workstation ADW4.5 of GE. The CBF map was standardized to the standard space *via* a standard ASL template (MNI152_3DASL.nii) using the MATLAB-based SPM12 toolbox. Because the CBF value obtained by 3D-ASL are affected by individual hemodynamic variation, we *Z*-transformed the registered CBF map through the DPABI (version 6.0) toolbox.

Pre-processing of rs-fMRI images using the MATLAB-based SPM8 and RESTplus V1.27 toolboxes included the following steps: (1) DICOM-NIFTI format transformation. (2) Remove of the first 10 time points. (3) Head movement correction to remove subjects with head movement displacement > 2 mm and head movement rotation > 2°. (4) Space standardization. (5) Removal of linear drift. (6) Regression with several covariates: the head motion parameters, noise from white matter signal and cerebrospinal fluid signal. (7) Bandpass filtering (0.01–0.08 Hz). The amplitude of low-frequency fluctuation (ALFF), fractional ALFF (fALFF), coherence-based regional homogeneity (Cohe-ReHo), and degree centrality (DC) maps were calculated for each subject, where ALFF, fALFF, and Cohe-ReHo were calculated before pre-processing with bandpass filtering, and a correlation coefficient of *r* = 0.25 was set when calculating the DC map. Finally, the Fisher *Z* transform was used to improve the normality of each indicator, and the results were smoothed using 6 mm × 6 mm × 6 mm Gaussian kernel.

Standard spatial masks of the bilateral hippocampus were obtained using the SPM8-based WFU PickAtlas toolkit. Then, masks were used to extract the value of CBF, ALFF, fALFF, Cohe-ReHo, and DC (positive weighted). The functional connectivity (FC) between the bilateral hippocampus and all voxels in the brain was calculated. Fisher *Z* transformation and smooth were performed too.

### 2.7. Statistics

Statistical analysis was conducted by version 25, (IBM Crop., Armonk, NY, USA). We used the independent two-sample *t*-test or non-parametric Mann–Whitney *U* test to compare the differences in demographic data and clinical laboratory indicators and neurocognitive test scores between the groups. The chi-square test was used to evaluate intergroup differences in gender. The significance level was set at *P* < 0.05. The bilateral hippocampal volumes, CBF, ALFF, fALFF, Cohe-ReHo, and DC (positive weighted) values partially did not follow the normal distribution. Therefore, we used a generalized linear model to analyze the differences in these imaging indicators between the two groups. In the bilateral hippocampal volume comparison, sex, age, years of education, and eTIV were used as covariates. In bilateral hippocampal CBF value comparisons, sex, age, and years of education were used as covariates. In bilateral hippocampal ALFF, fALFF, Cohe-ReHo, and DC (positive weighted) value comparisons, sex, age, years of education, and mean head movement parameters were used as covariates.

Independent two-sample *t* test was applied to the FC of bilateral hippocampus between two groups using the RESTplus V1.27 toolbox, using the built-in Brain Mask (61 × 73 × 61) as a template, with sex, age, education, and mean head movement parameters as covariates. Gaussian random field (GRF) correction was performed (voxel-level threshold of *P* < 0.001 and cluster-level threshold of *P* < 0.05, two-tailed).

The cognitive functional scores, clinical indicators and some imaging indicators did not follow the normal distribution. Therefore, a generalized linear model was used to obtain the residuals of the AVLT (immediate) score, CDT score, MoCA score, GPT (R), GPT (L) elapsed time, and right hippocampus CBF after the regression of sex, age, years of education. Then we obtained residuals of bilateral hippocampal volumes after the regression of sex, age, years of education and eTIV. As well as we obtained residuals of bilateral hippocampus ALFF, fALFF, and Cohe-ReHo after the regression of sex, age, years of education and mean head motion parameters. Spearman correlation analysis was used to calculate the correlations among clinical laboratory indicators (HbA1c, FINS, FBG, and HOMA-IR), cognitive tests [residuals of AVLT (immediate) score, CDT score, MoCA score, GPT (R), and GPT (L) elapsed time] and imaging indicators (residuals of bilateral hippocampal volume, ALFF, fALFF, and Cohe-ReHo values, and right hippocampal CBF value).

## 3. Results

### 3.1. Demographic data and clinical laboratory indicators

There were no significant differences in sex, age, years of education, systolic pressure (SBP) or diastolic pressure (DBP) between two groups. Demographic information and clinical laboratory indices are summarized in Table 1.

**TABLE 1:** Demographic data and clinical biochemical indicators of all subjects.

	T2DM (*n* = 30)	HCs (*n* = 45)	Statistics (*t*, *x*^2^, *z*)	*P* value*
Age (years)	50.37 ± 8.72	48.67 ± 7.13	0.924	0.358
Sex (male/female)	19/11	24/21	0.736	0.391
Education (years)	12.00 (9.00; 12.75)	9.00 (6.00; 12.00)	−0.695	0.487
SBP (mmHg)	120.57 ± 10.07	118.00 (112.00; 124.00)	−1.343	0.179
DBP (mmHg)	79.43 ± 9.18	78.00 (75.00; 80.00)	−0.824	0.410
HbA1c (%)	8.70 (7.10; 10.28)	N/A	N/A	N/A
FINS (μIU/ml)	8.01 (7.12; 9.06)	N/A	N/A	N/A
FBG (mmol/L)	7.33 (5.48; 11.48)	N/A	N/A	N/A
HOMA-IR	2.51 (1.95; 3.79)	N/A	N/A	N/A

SBP, systolic pressure; DBP, diastolic pressure; FINS, fasting insulin; FBG, fasting blood glucose; HOMA-IR, homeostatic model assessment for insulin resistance; N/A, not applicable. *P < 0.05.

### 3.2. Neurocognitive test scores

The AVLT (immediate) score (*P* = 0.019), CDT score (*P* = 0.015), and MoCA score (*P* = 0.008) of the T2DM group were significantly lower than those of the HCs. The performance of GPT (R) (*P* = 0.006) and GPT (L) (*P* = 0.012) in T2DM group were worse than those of the HCs. The results of the cognitive tests are shown in Table 2.

**TABLE 2:** Neuropsychological result of two groups.

	T2DM (*n* = 30)	HCs (*n* = 45)	Statistics (*z*)	*P* value
AVLT (immediate)	19.00 (17.00; 23.25)	24.00 (18.00; 27.00)	−2.342	0.019*
AVLT (5 min)	8.00 (6.00; 9.00)	8.00 (7.00; 10.00)	−0.593	0.553
AVLT (20 min)	9.00 (6.75; 10.00)	8.00 (7.00; 9.00)	−0.587	0.557
AVLT (recognition)	11.50 (9.00; 12.00)	11.00 (10.00; 12.00)	−0.541	0.588
DST (forward)	8.00 (6.75; 9.00)	8.00 (8.00; 9.00)	−1.621	0.105
DST (inverse)	4.00 (3.00; 5.00)	4.00 (3.00; 4.50)	−1.052	0.293
CDT score	3.00 (2.00; 3.00)	3.00 (3.00; 3.00)	−2.428	0.015*
MoCA score	26.00 (24.50; 27.00)	27.00 (26.00; 28.50)	−2.649	0.008*
GPT (R) (s)	83.40 (71.70; 99.12)	75.00 (66.30; 82.00)	−2.770	0.006*
GPT (L) (s)	89.77 (78.00; 107.83)	80.00 (75.00; 90.50)	−2.499	0.012*

AVLT, auditory verbal learning test; DST, the digital span test; CDT, the clock drawing test; MoCA, Montreal cognitive assessment; GPT, grooved pegboard test. *P < 0.05.

### 3.3. Bilateral hippocampal imaging indicators

**FIGURE 1:**
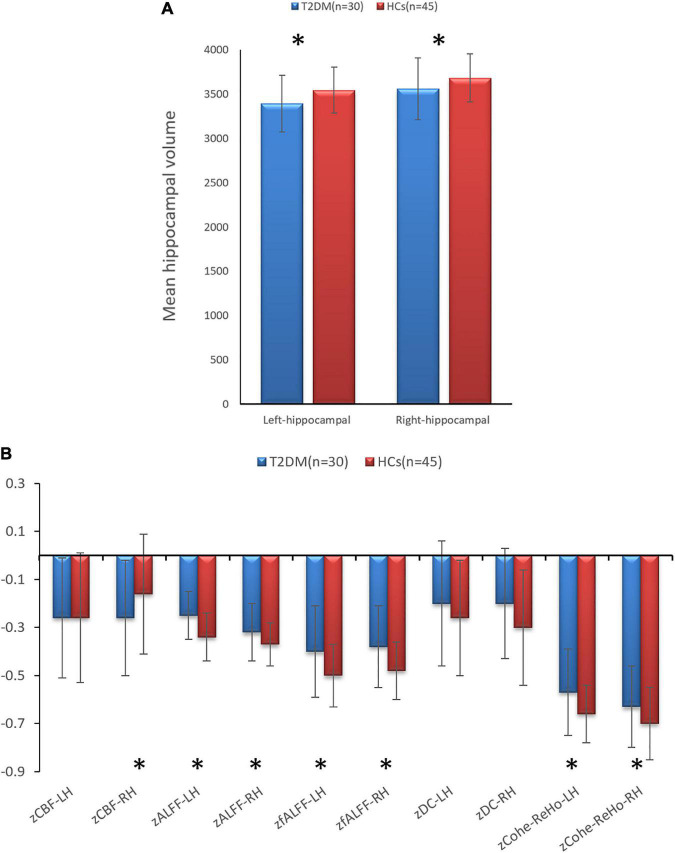
**(A)** Comparison of the volumes of bilateral hippocampal between type 2 diabetes mellitus (T2DM) and healthy controls (HCs). **(B)** Comparison of the imaging indicators of bilateral hippocampal between T2DM and HCs. *P < 0.05.

In the T2DM group, the bilateral hippocampal volume (left, *P* = 0.001; right, *P* = 0.015) and right hippocampal CBF value (*P* = 0.021) were lower than those in HCs. The bilateral hippocampal ALFF value (left, *P* < 0.001; right, *P* = 0.018), fALFF value (left, *P* = 0.005; right, *P* = 0.002), and Cohe-ReHo value (left, *P* = 0.003 right, *P* = 0.038) of the T2DM group were higher than those of HCs. They were shown in Figure 1 and Table 3.

**TABLE 3:** Bilateral hippocampal imaging indicators.

	T2DM (*n* = 30)	HCs (*n* = 45)	Difference and 95% execution interval	Statistics (*t*, *z*, *wald x^2^*)	*P* value
eTIV (mm^3^)	1,484,735.77 ± 113,576.88	1,481,841.90 ± 111,408.51	N/A	0.109	0.913
Mean head motion parameters	0.07 (0.05; 0.09)	0.06 (0.05; 0.09)	N/A	−1.168	0.243
Volume-LH (mm^3^)	3,396.30 ± 319.75	3,546.75 ± 259.53	−156.60 (−245.86; −67.34)	11.823	0.001*
Volume-RH (mm^3^)	3,561.89 ± 349.79	3,683.69 ± 271.83	−122.42 (−221.45; −23.40)	5.872	0.015*
*z*CBF-LH	−0.26 ± 0.25	−0.26 ± 0.27	−0.01 (−0.13; 0.11)	0.043	0.836
*z*CBF-RH	−0.26 ± 0.24	−0.16 ± 0.25	−0.13 (−0.24; −0.02)	5.310	0.021*
*z*ALFF-LH	−0.25 ± 0.10	−0.34 ± 0.10	0.09 (0.04; 0.13)	14.506	<0.001*
*z*ALFF-RH	−0.34 (−0.38; −0.25)	−0.37 ± 0.09	0.05 (0.01; 0.1)	5.610	0.018*
*z*fALFF-LH	−0.40 ± 0.19	−0.50 (−0.58; −0.40)	0.09 (0.03; 0.16)	7.873	0.005*
*z*fALFF-RH	−0.38 ± 0.17	−0.50 (−0.58; −0.40)	0.09 (0.03; 0.16)	9.269	0.002*
*z*DC-LH	−0.20 ± 0.26	−0.28 (−0.39; −0.18)	0.06 (−0.05; 0.17)	1.302	0.254
*z*DC-RH	−0.26 (−0.36; −0.10)	−0.30 (−0.43; −0.23)	0.09 (−0.02; 0.20)	2.808	0.094
*z*Cohe-ReHo-LH	−0.61 (−0.69; −0.49)	−0.67 (−0.76; −0.59)	0.10 (0.03; 0.16)	8.781	0.003*
*z*Cohe-ReHo-RH	−0.68 (−0.73; −0.57)	−0.74 (−0.79; −0.64)	0.07 (0.004; 0.15)	4.305	0.038*

eTIV, estimated total intracranial volume; CBF, cerebral blood flow; LH, left hippocampal; RH, right hippocampal; ALFF, amplitude of low-frequency fluctuation; fALFF, fractional ALFF; Cohe-ReHo, coherence-based regional homogeneity; DC, degree centrality. *P < 0.05.

### 3.4. Analysis of bilateral hippocampal and whole-brain functional connectivity

There is no significant difference for FC between two groups.

### 3.5. Correlation between clinical laboratory indicators and neurocognitive test scores

**FIGURE 2:**
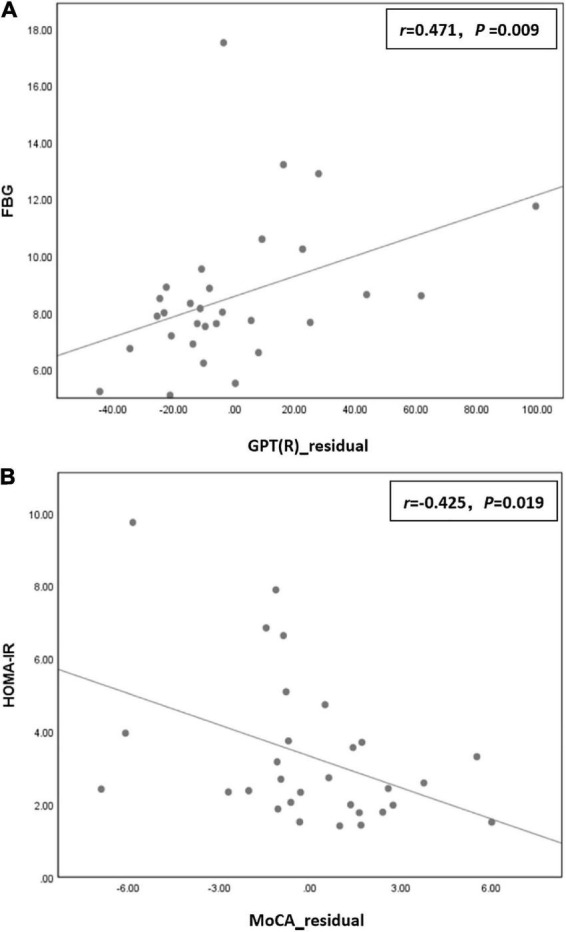
**(A)** The residual of grooved pegboard test (GPT) (R) time in the type 2 diabetes mellitus (T2DM) group was positively correlated with fasting blood glucose (FBG) (*r* = 0.471, *P* = 0.009) in the T2DM group. **(B)** The residual of the Montreal cognitive assessment (MoCA) score was negatively correlated with homeostatic model assessment for insulin resistance (HOMA-IR) (*r* = –0.425, *P* = 0.019) in the T2DM group.

Correlation analysis showed that the residual of GPT (R) time in the T2DM group was positively correlated with FBG (*r* = 0.471, *P* = 0.009), and the residual of the MoCA score was negatively correlated with HOMA-IR (*r* = −0.425, *P* = 0.019; Figure 2).

### 3.6. Correlation between clinical laboratory indicators and imaging indicators

**FIGURE 3:**
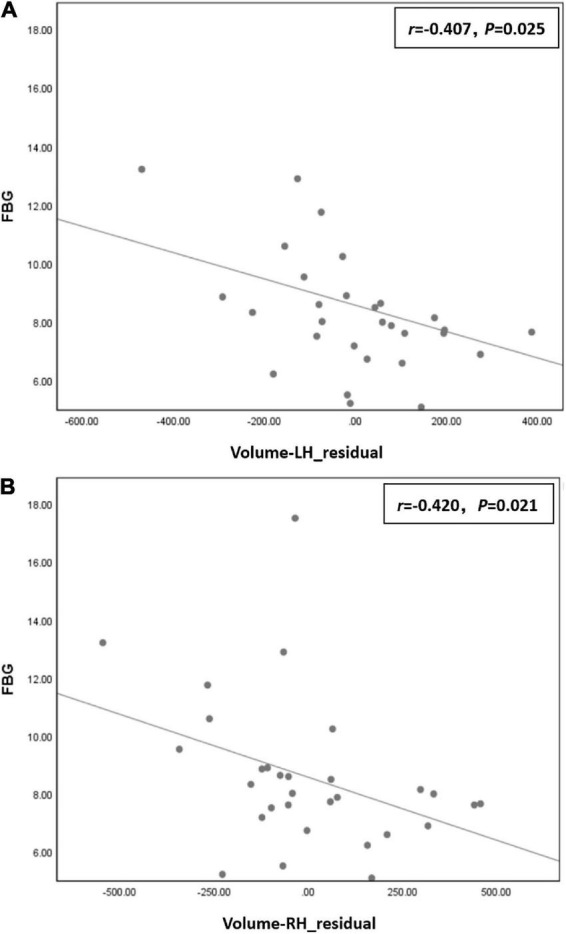
**(A)** Fasting blood glucose (FBG) was negatively correlated with residual of left hippocampal volume (*r* = –0.407, *P* = 0.025) in the type 2 diabetes mellitus (T2DM) group. **(B)** FBG was negatively correlated with residual of right hippocampal volume (*r* = –0.420, *P* = 0.021) in the T2DM group.

Correlation analysis showed that FBG was negatively correlated with residuals of left hippocampal volume (*r* = −0.407, *P* = 0.025) and right hippocampal volume (*r* = −0.420, *P* = 0.021) in the T2DM group (Figure 3).

### 3.7. Correlation between neurocognitive test scores and imaging indicators

**FIGURE 4:**
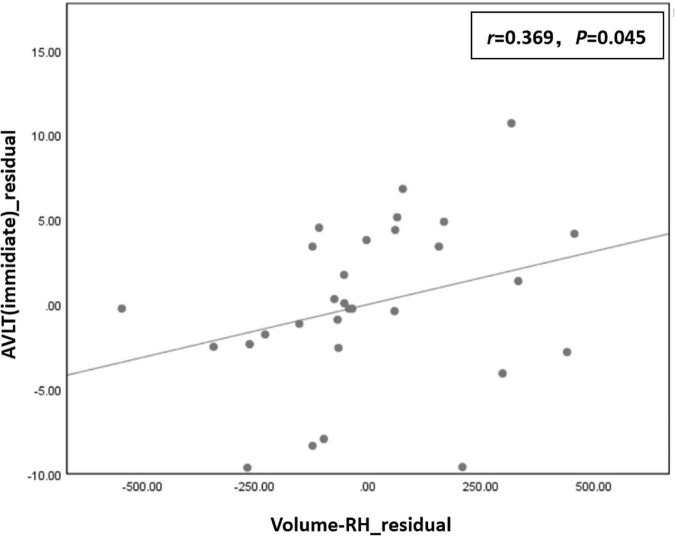
The residual of the auditory-verbal learning test (AVLT) (immediate) score was positively correlated with the residual of right hippocampal volume (*r* = 0.369, *P* = 0.045) in the type 2 diabetes mellitus (T2DM) group.

Correlation analysis showed that the residual of the AVLT (immediate) score was positively correlated with the residual of right hippocampal volume (*r* = 0.369, *P* = 0.045) in the T2DM group (Figure 4).

## 4. Discussion

In the present study, the T2DM group performed worse on the AVLT (immediate), CDT, MoCA, GPT (R), and GPT (L) than the HC group. The AVLT is mainly used to assess subjects’ memory. Memory is the most frequently studied and reported cognitive domain in T2DM. A meta-analysis has proposed that the reduced immediate memory capacity of T2DM may be a longitudinal process starting with pre-diabetes. The possible explanation for the effect of T2DM on memory is that the inflammatory mediators gradually affect microvascular and macrovascular structure, resulting in reduced memory capacity (Sadanand et al., 2016). In this study, we also found that the immediate memory of the T2DM group was worse than that of HCs. However, the delayed memory ability was not significantly different between the two groups, probably because the two groups were younger (the mean age of the T2DM group was 48.67 ± 7.13 and the mean age of the HCs was 50.37 ± 8.72). The CDT is mainly used to evaluate subjects’ cognition, visuospatial, and executive function. The GPT is mainly used to assess subjects’ executive function and neuromotor flexibility. Our findings suggest that the T2DM group had worse executive function than the HC group, which is consistent with earlier studies on executive dysfunction in patients with T2DM (Qiu et al., 2006). The MoCA is mainly used for a brief evaluation of cognitive functions such as memory, visuospatial, abstract thinking, attention, and executive function. Many previous studies have shown that cognitive function is impaired in T2DM patients, mainly manifested by decreased information processing ability, loss of memory and attention, disruption of executive function and visuospatial ability (Hoogenboom et al., 2014; Macpherson et al., 2017). Evidence from studies suggested an age-related increase in 1.5–2-times cognitive decline with the onset of T2DM (Cukierman et al., 2005; Biessels et al., 2006). In the available literature, a significant decline in neurocognitive function of T2DM patients has been attributed to accelerated cognitive aging (Messier, 2005). Brands et al. (2007) found that T2DM patients performed significantly worse than HCs in attention, executive function, processing speed, and memory, suggesting that T2DM accelerated cognitive decline in several cognitive domains. On the other hand, Messier (2005) pointed out that episodic memory and processing speed were the first domains to be affected in T2DM and then expanded to other domains with age.

Moreover, the correlation analysis in this study showed that the residual of GPT (R) time was positively correlated with FBG, and the residual of MoCA score was negatively correlated with HOMA-IR in the T2DM group, indicating that cognitive decline in T2DM patients was closely related to hyperglycemia status and insulin resistance. The hyperglycemic state leads to neuronal dysfunction and apoptosis through the polyol pathway, increased advanced glycation end products, increased lipid peroxidation, and oxidative stress (Sima et al., 2004). In addition, chronic hyperglycemia is one of the main causes of vascular dysfunction and impairment in T2DM patients. Cerebrovascular diseases caused by chronic hyperglycemia can reduce CBF and lead to ischemia and hypoxia which eventually lead to the apoptosis of nerve cells, thus affecting the cognitive function of patients. Central system insulin resistance leads to the reduction of neurocognitive function in T2DM patients by affecting the function of insulin in the brain. In summary, we hypothesized that hyperglycemic status and central insulin resistance may be important reasons for the reduced neurocognitive function in T2DM patients.

In terms of structure and perfusion, this study found that the T2DM group’s CBF of the bilateral hippocampus and right hippocampus were lower than that in the HCs, and FBG was negatively correlated with bilateral hippocampal volume in the T2DM group. Previous studies have found hippocampal atrophy even in pre-diabetes patients (Convit et al., 2003). A review in the Lancet (McCrimmon et al., 2012) described learning and memory deficits and hippocampal atrophy as abnormalities that clearly distinguish between T2DM and type 1 diabetes mellitus, but the reason for the more severe hippocampal atrophy in T2DM remains unclear. However, the model of diabetic tissue damage proposed by Brownlee (2001) shows that chronic hyperglycemia is deleterious to microvascular endothelial cells, thus compromising glucose transport across the blood brain barrier. At the same time, cerebrovascular disease caused by chronic hyperglycemia can reduce CBF. In this study, we also confirmed that T2DM patients have decreased blood perfusion in hippocampus. It is possible that hippocampal ischemia and hypoxia may eventually lead to apoptosis of neuronal cells, resulting in decreased hippocampal volume. On the other hand, central insulin has been shown to adjust synaptic initiation and remodeling (Abbott et al., 1999). As insulin was previously revealed to boost dendritic spine formation in rat hippocampal neurons. Conversely, the use of closed antibodies or downregulation of IR signaling can result in reduced dendritic spines (Lee et al., 2011). It has also been shown that insulin can promote the axonal outgrowth of fetal brain cells (Schechter et al., 1996). However, a correlation between hippocampal volume and HOMA-IR was not found in this study. This may be that the patient’s medication has an effect on insulin resistance. But in this study, different T2DM patients used different hypoglycemic drugs and traditional Chinese medicine treatment. In the future, better experimental design is still needed to incorporate the effects of drugs on experiments.

Regarding brain function, this study showed that the ALFF, fALFF, and Cohe-ReHo of the bilateral hippocampus were higher in T2DM group compared to HCs. After GRF multiple comparison correction, the comparison of FC between the two groups was negative. ALFF and fALFF reflect the situation of local spontaneous neural activity in the brain, while Cohe-ReHo reflect the degree of coordination of local neural activity. The results of this study demonstrated increased local spontaneous neural activity and coordination in the hippocampus in T2DM. Previous studies have also shown enhanced FC between the left hippocampus and the left inferior frontal/left inferior parietal lobe in T2DM group. The researchers suggest that adaptive compensatory mechanisms for brain function in the early stages of T2DM may counteract the underlying cognitive decline (Fang et al., 2019). This additional recruitment of brain activation was seen as a compensatory mechanism in the brain in the early stages of cognitive impairment, which is more commonly in studies of Alzheimer’s disease (AD). Dickerson et al. (2005) found that compared to HCs, MCI group showed greater hippocampal activation, but AD group showed hypofunction and atrophy in the hippocampus and entorhinal cortex. Thus, this suggests that there is an early stage of increased activation of the medial temporal lobe in AD. Studies found that MCI patients showed an enhanced activation in posterior hippocampus, parahippocampal gyrus, and fusiform gyrus compared with HCs, while voxel-based morphometry (VBM) analysis revealed atrophy in the anterior part of the left hippocampus in the MCI group. The investigators hypothesized that the increased activation in the posterior medial temporal lobe and tightly connected fusiform gyrus in MCI patients which is a compensatory mechanism for early atrophy in the anterior medial temporal lobe (Hämäläinen et al., 2007). There were also some studies showing that compensatory hyperactivation was viewed as an imaging marker for predicting AD (Dickerson et al., 2004; O’Brien et al., 2010). In the follow-up study of Miller et al. (2008), subjects varied greatly in the degrees and rates of cognitive decline, and subjects with greater hippocampal activation experienced increased degrees and rates of subsequent cognitive decline. Therefore, our results may demonstrate that the increase in hippocampal neural activity and regional coordination is an adaptive compensation for cognitive decline in T2DM patients, but whether this compensatory increase in the hippocampus in T2DM patients can predict future cognitive dysfunction will require further follow-up studies to draw evidence. At the same time, we did not find a changed FC between the hippocampus and whole-brain in T2DM. We speculated that this may be due to the non-linear dynamic changes in the activity of the hippocampus during the course of the disease, but it should be confirmed by further studies in the future.

Furthermore, the correlation analysis in the present study showed that the AVLT (immediate) score was positively associated with the right hippocampal volume in the T2DM group. It has been previously shown that decreased immediate memory in T2DM is associated with hippocampal atrophy (Gold et al., 2007). In previous reports, Milner and Klein (2016) emphasized the important role of the hippocampus in establishing new lasting memory and memory consolidation, and damage to the hippocampus leads to the loss of recent memory in patients. However, in this study, only an AVLT (immediate) score reduction was found, while the delayed recall score was not significantly different between the two groups. Combined with the increase in local spontaneous neural activity and coordination in the hippocampus of the T2DM group, we speculate that this compensatory mechanism makes an adaptive compensation for the delayed recall function in T2DM patients, or the AVLT test alone may not specifically reflect the delayed recall status. The change in delayed recall function in T2DM patients still needs further investigation in the future.

## 5. Limitations

Firstly, this is a cross-sectional study, and longitudinal follow-up will be conducted in the future to explore the dynamics of the hippocampal subregion. Secondly, because of the insidious onset of type 2 diabetes, the course of disease was not recorded in this study. In the future, appropriate methods will be needed to incorporate the course of disease into research. Thirdly, because different T2DM patients used different hypoglycemic drugs, we could not evaluate the effects of drugs on the brain. In the future, better experimental design is still needed to incorporate the effects of drugs on experiments.

## 6. Conclusion

This study indicate that the volume and perfusion of the hippocampus were decreased in T2DM patients that may be related to chronic hyperglycemia. Reduced bilateral hippocampal volume is associated with loss of immediate memory in T2DM patients. Local spontaneous neural activity and coordination are increased in the hippocampus of T2DM patients, possibly as an adaptive compensation for cognitive decline.

## Data availability statement

The raw data supporting the conclusions of this article will be made available by the authors, without undue reservation.

## Ethics statement

The studies involving human participants were reviewed and approved by the Medical Research Ethics Committee of Guangzhou University of Chinese Medicine (approval number: K2020115), and written informed consent was obtained from all subjects.

## Author contributions

ML and YFL designed this study and wrote the manuscript. KZ contributed to the statistical analysis. XT administered the neuropsychological tests. YC did the data analysis and amended the manuscript. CQ, SQ, and YL are the guarantors of this study and chiefly responsible for the whole process of the experiment. All authors contributed to the article and approved the submitted version.
